# Update on the epidemiology and treatment of eating disorders among older people

**DOI:** 10.1097/YCO.0000000000000893

**Published:** 2023-07-19

**Authors:** Barbara Mangweth-Matzek, Kai K. Kummer, Hans W. Hoek

**Affiliations:** a University Hospital of Psychiatry II, Department of Psychiatry, Psychotherapy, Psychosomatics and Medical Psychology; b Institute of Physiology, Medical University of Innsbruck, Innsbruck, Austria; c Parnassia Psychiatric Institute, The Hague; d Department of Psychiatry, University Medical Center Groningen, University of Groningen, Groningen, The Netherlands; e Department of Epidemiology, Columbia University, Mailman School of Public Health, New York, USA

**Keywords:** eating disorders, midlife, older men, older women, treatment

## Abstract

**Purpose of review:**

We reviewed the recent literature on the epidemiology and treatment of eating disorders among middle-aged and older women and men.

**Recent findings:**

Recent studies show that among older female persons, the prevalence rates with full diagnoses of eating disorders based on DSM-IV or DSM-5 criteria are between 2.1 and 7.7%, and among older men less than 1%. These studies show that the prevalence of eating disorders decreases by age in women, but it does not get towards zero even in very high age. Middle age, with a peak around 50, is also a critical time for the occurrence of eating disorders in men. Women who reported severe menopausal symptoms showed more eating disorder pathology compared with those with low symptoms during menopausal transition.

**Summary:**

Eating disorders do occur in middle and older age of both sexes. Shame and stigmatization have decreased, and medical awareness and explicit assessment of eating behavior in all age groups have developed. What puberty is for eating disorders in adolescence and young age is menopausal transition for midlife women. Also in men, associations with hormonal disturbances are possible. Treatment approaches should consider treatment strategies tailored to older women and men, addressing the context of midlife and aging.

## INTRODUCTION

Many studies show that eating disorders do occur in people of all ages and genders and are not limited to women of young age [1–6]. In a previous issue of this journal, we have reviewed the literature on epidemiology and treatment aspects of eating disorders in middle-aged and older women and men till 2017 [7]. In this article, we give an update of the recent literature on eating disorders and disordered eating in the context of epidemiology and treatment in older people between June 2017 and May 2023. We searched for articles using PubMed, Ovid MEDLINE(R), EMBASE, and PsycINFO, based on the terms ‘eating disorders’, ‘eating behavior’, ‘disordered eating’, ‘epidemiology’, ‘prevalence rate’, ‘menopause’, ‘body image’, ‘severe and enduring eating disorder’, ‘treatment’, ‘midlife’, ‘middle age’, ‘older age’, ‘women’, ‘men’, ‘females’, and ‘males’. 

**Box 1:**
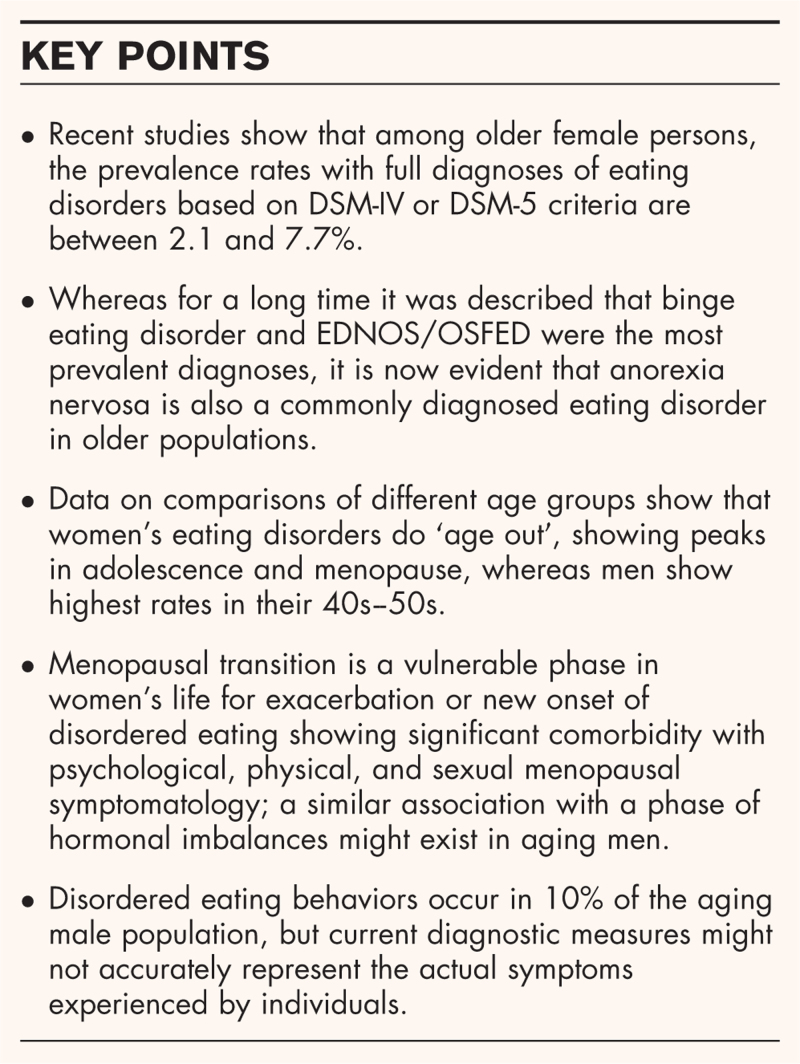
no caption available

## DIAGNOSES AND ASSESSMENT

Whereas the first studies on older women were based on case reports and small samples [8–10], recent studies and reviews describe larger sample sizes including ages mostly above 40 till 80 years [11–14], such as a good review article on disordered eating and eating disorders in midlife and older women by Samuels *et al.*[11] in 2019.

As already discussed in our previous review (2017) [7], diagnoses of eating disorders are still based on three different levels: full diagnoses of eating disorders (according to International Classification of Diseases (ICD) or Diagnostic and Statistical Manual of Mental Disorders (DSM)) including anorexia nervosa, bulimia nervosa, binge eating disorder (BED), and OSFED (Other Specified Feeding and Eating Disorder); core eating disorder symptoms including BMI below 18.5, binge eating with and without purging (defined by ICD or DSM criteria); and scores of standardized questionnaires assessing disordered eating or risk of eating disorders. Recent studies show a predominance of eating disorder assessment based on ‘nonfull’ diagnoses as described above [13,14]. This mirrors the fact that most of the recent studies are community studies [12–14,15^▪^,^16^^▪▪^,17^▪^] using a questionnaire-based symptom assessment rather than a face-to-face assessment. Older persons with eating disorders are hardly seen in specialized care, and there are still almost no clinical studies on eating disordered patients in middle or older age, although it is empirically evident that eating disorders and disordered eating do occur in these cohorts [4,7,11]. One major factor that people with eating disorders often do not seek specific eating disorder treatment might be the intertwining with aging-related symptoms and stigmatization and shame.

## EPIDEMIOLOGY AND TREATMENT IN WOMEN

Since our review from 2017 [7], some interesting new studies have focused on symptomatology and important aspects of pathological eating in women.

### Symptomatology

Data on middle-aged and older women show that from the various full diagnoses of eating disorders based on DSM-5 [18] and DSM-IV [19], OSFED or EDNOS (Eating Disorder Not Otherwise Specified) and BED are the most prevalent diagnoses [5,16^▪▪^,^20^–^22^]. Brown *et al.*[14] showed atypical anorexia nervosa as the most prevalent diagnoses of women in their 40s and 50s, similar to Presskreischer *et al.*[16^▪▪^]. Corresponding to the findings of full diagnoses, binge eating is described as the most prevalent core eating disorder symptom [2,23^▪^,^24^]. Standardized questionnaires assess various traits associated with eating disorders, such as restrictive eating, shape concern, food preoccupation, and so forth, and often use a cut-off in order to assess disordered eating or the risk for an eating disorder [25,26].

### Prevalence rates

It is still very difficult to assess exact prevalence rates of any eating disorders or the various diagnoses because of the small number of studies, the different sample characteristics, assessment instruments, methodological approaches, different age groups and genders. Therefore, presented numbers should be received as trend ranges rather than exact numbers. Further, it is obvious that full diagnoses reach lower prevalence rates compared with single-symptom diagnoses or cut-off rates from questionnaires.

Recent studies show that among older persons, the prevalence rates with full diagnoses of eating disorders based on DSM-IV or DSM-5 criteria [5,16^▪▪^,^20^–^22^] are between 2.1 and 7.7%, of core eating disorder symptoms with binge eating only as the dominant symptom followed by binging and purging [2,23^▪^,^24^] range between 3.8 and 9%, and of disordered eating or risk for eating disorders based on standardized questionnaires [12,13,23^▪^,^24^–^26^] are between 2.6 and 16%.

In an older study, Preti *et al.*[21] compared different age groups using similar methodology regarding eating disorder pathology; they looked at eating disorders in a subsample of 4139 women and men of six different European countries, on average aged 47.1 years. They described a life-time risk of DSM-IV eating disorders including anorexia nervosa, bulimia nervosa, and BED in 5.12% of their age cohort of 18–29 years, 2.38% of the 30–44 years old group, and 1.29% in their 45+ cohort. Unfortunately, there were no gender-specific results in relation to different age cohorts. Hadjigeorgiou *et al.*[26] used the Eating Attitude Test EAT-26 [27] and found scores equal to or above 20 indicating risk for eating disorder in 16% of their 268 adolescents, 23% in their 596 young adults, and 22% in their 130 women of middle age.

Presskreischer *et al.*[16^▪▪^] conducted an important large-scale cross sectional study on prevalence rates of eating disorders among people enrolled in Medicare in the USA. Medicare is a federal insurance program that covers adults aged 65 and older, and provides coverage for qualifying individuals of any age with disabilities. The sample included 11 962 287 Medicare enrollees of whom 0.15% had an eating disorder diagnosis. Compared with those without an eating disorder diagnosis, a greater proportion of individuals with an eating disorder were female individuals (73.8 vs. 54.3%), under age 65 (41.6 vs. 15.5%), and dually eligible for Medicaid because of disability or low-income qualification (48 vs. 19.6%). Individuals with eating disorders had higher rates of comorbid conditions, with the greatest differences in cardiac arrhythmias (35.3 vs. 19.9%), arthritis (40.1 vs. 26.6%), and thyroid conditions (32.2 vs. 19.4%). Spending was four times higher for enrollees with eating disorders compared with those without overall ($29 456 vs. $7418).

Another important study on a 30-year longitudinal course of eating disorder pathology was performed by Brown *et al.*[14]. Participants were 900 college students (624 women and 267 men) aged around 20 years who were examined using the Eating Disorder Inventory (EDI) [28] and single eating disorder symptom questions based on DSM-III-R and DSM-5, respectively, for assessments every 10 years, resulting in a participation rate of around 70% at the 30-year follow-up. They found that the prevalence rate of any eating disorder in women decreased from 19.4% in women aged 20 years to 10.8% in women aged 30 years to 8% in women aged 40 and 50 years, respectively. This course in female individuals differs significantly from similar age groups in male individuals, showing no changes in point prevalence over the years and no gender difference by age 50 [14]. In addition, they found that almost 30% of their cases reported an onset in older age. Further, Mitchell *et al.*[15^▪^] examined US military veterans (*N* = 1187 aged on average in the 50s) using self-report measures including the Eating Disorder Examination Questionnaire (EDE-Q) [29]. They found high prevalence rates (32%) based on the cut-off score of 2.8 among women. The peak of disordered eating was in the age group 40–49, followed by significant decreases in the age group at least 60 years.

Research of disordered eating and eating disorders, respectively, in different age cohorts is important in order to assess courses over time and identify possible vulnerable phases and gender differences. Thus, the presented studies show that the prevalence of eating disorders decreases by age, but it does not get towards zero even in high age [20,30].

### Menopause

What puberty is for eating disorders in adolescence and young age is menopausal transition for midlife. Research on the association between eating disorder pathology and menopause is young and sparse. It was our group that first described significantly higher rates of eating disorders in perimenopausal women compared with premenopausal or postmenopausal women [31]. This study initiated further studies on the topic of menopause and eating, resulting in findings that are still controversial. There are studies confirming an association of specific menopausal stages and disordered eating [32–34] and studies that did not find such an association [23^▪^,^24^,^35^]. A recent study added a new focus to the topic. Mangweth-Matzek *et al.*[23^▪^,^24^] investigated not only the connection between various menopausal stages and eating behavior but also the relationship between menopausal symptomatology and eating behavior. Their results showed no significant differences in the prevalence rate of eating disorder symptoms (varying between 5 and 9%) among the various menopausal stage groups, but striking differences (*P* < 0.001) between women describing high menopausal symptomatology vs. those with low symptomatology assessed by the Menopausal Rating Scale (MRS) [36]. Thus, women who reported lots of menopausal symptoms showed more eating disorder pathology compared with those with low symptoms during menopausal transition. The rationale behind these findings is two-fold: menopausal stages, especially perimenopause, lacks specific biological markers for exact diagnosis and, therefore, rating overlaps do occur; and not all women suffer menopausal symptoms during the transition, and it seems obvious that those experiencing various complaints are also more vulnerable for developing disordered eating.

### Severe and enduring eating disorders

There are still no definitive criteria for severe and enduring eating disorders (SEED) and severe and enduring anorexia nervosa (SE-AN), studies are still sparse, but data clearly state that the treatment of patients with chronic eating disorder should differ from treatment of those with short duration [37,38]. There are also other terms like longstanding, complex eating disorder [39], or long-lasting anorexia nervosa [40] that describe this chronic group of patients. Hay and Touyz [41] in 2018 have given three criteria to define SE-AN [42]:

(1) A persistent state of dietary restriction, underweight, and overvaluation of weight/shape with functional impairment;
(2) Duration of >3 years of anorexia nervosa; and
(3) Exposure to at least two evidence-based treatments appropriately delivered together with a diagnostic assessment and formulation that incorporates an assessment of the person's eating disorder health literacy and stage of change.

It is evident that severe and enduring eating disorders can affect both young and older women. Those older women are between 40 and 80+ years, have an onset of the eating disorder most often in adolescence but in one-third of the cases later in life [11], describe an eating disorder duration of 10, 20, 30 or even at least 40 years [11,31] and are often characterized by chronological symptom persistence. So far, there are no explicit data of SEED/SE-AN or long-lasting eating disorder in cohorts of middle or older age. The interaction of chronic eating disorder with age-depending illnesses will create a very special, medical and therapeutic challenge because of the denial behavior and noncompliance of many of the chronic eating disordered patients. Further research is needed in order to assess various aspects of this subcategory of eating disorders in older age.

### Treatment

Although, it is reported that the admission rate of eating disordered women aged 40+ in inpatient and outpatient treatment facilities has increased in the last two decades [11,40,43], studies on treatment of middle-aged or older patients with eating disorders are rare [40,43,44^▪▪^,^45^] and predominantly based on case reports [44^▪▪^,^46^].

Mulchandani *et al.*[44^▪▪^] did a valuable, interesting review on treatment of eating disorders in older people, including 35 articles based on 39 cases. The participants were aged between 66 and 94 years, of whom 85% were female individuals. The most prevalent diagnosis was anorexia nervosa, and almost 60% reported an onset of the disorder after age 40. Ninety-five percent received a treatment mostly based on a multidimensional approach including psychotherapy and pharmacotherapy. Eighty percent of the cases who were treated reported improvement of the eating disorder.

Gaudiani *et al.*[46] described three cases of patients above age 30 years with terminal anorexia nervosa focusing on the high mortality rate of chronic anorexia nervosa. Although not a recent study but valuable, Ballard and Crane [45] examined 1137 outpatients, 93% female individuals, comparing various age groups (15–75+). They found that treatments of individuals aged 44–55 years were less expensive than for 15–24 years. Individual therapy was the most common treatment modality; however, younger patients also received family therapy. Middle-aged patients had higher drop-out rates and lower return-to-care rates [45].

Marzola *et al.*[40] investigated duration of SE-AN in 169 inpatients (92% female individuals) and found that long-duration-illness (≥7 years) characterized middle-aged and older patients with mostly binge-purge anorexia nervosa-subtype and many treatment failures before. Their data showed that enduringness of anorexia nervosa was not a specifier of severity of symptomatology and hospitalization was effective for them including various aspects: reduction of life-threatening conditions; strengthening of motivation for therapy; symptom management; psychological work on possible causes of admission; and psychoeducation for families [40].

Existing data show clearly that treatment helps eating disordered patients of middle and older age.

## EPIDEMIOLOGY AND TREATMENT IN MEN

For eating disorders in male individuals, the body of literature is even more limited, with even fewer studies focusing on older men. Although research in this direction has increased over the last 10–20 years, the first systematic review on eating disorders in aging male individuals by Reas and Stedal [6] was only published in 2015. Since our last review [7], only a few studies have further focused on the occurrence of disordered eating and eating disorders in male individuals.

### Prevalence rates

The prevalence of eating disorders in male individuals might be strongly underestimated because of the paucity of empirical research data and the lack of sensitivity of assessment tools for this population [47]. The range of reported prevalence rates is broad and barely covers DSM-5 diagnoses, especially for different age groups [48]. While in a Swiss cohort of men aged 15–60, lifetime prevalence of anorexia nervosa, bulimia nervosa and BED was 0.02, 0.9 and 0.7%, respectively, prevalence of subthreshold BED was with 1.6% almost twice as high than in women [49]. In a US self-report study focusing specifically on BED, lifetime prevalence in male individuals was reported at 1.41% for DSM-5 diagnosis compared with 0.92% by applying DSM-IV-TR criteria [50].

### Symptomatology

In 2016, we published one of the first reports on an Austrian community sample of aged male individuals, which showed a prevalence rate of 7% for disordered eating based on EDE-Q criteria [51]. In a recent follow-up study on men of different ages visiting fitness centers, we found that 10% of respondents met criteria for current eating disorder symptoms, most with regard to binge eating, and older men between 60 and 80 years showed similar EDE-Q scores as younger men [52^▪^]. Middle-aged men showed the highest scores on the EAT-26 questionnaire, as well as on the subscales ‘dieting’ and ‘oral control’ in the male population of a cross-sectional study in Cyprus [26]. Although decreasing with age, still 29.4% of the 46–60 year old men indicated to exercise more than 60 min per day to lose or to control weight.

The previously mentioned longitudinal study by Brown *et al.*[14] also covered male participants. The prevalence of eating disorder diagnoses remained stable over the 30-year observation period in men, while it decreased in women, and point prevalence of new-onset eating disorder cases was not different between sexes at age 50. Interestingly, while the drive for thinness decreased over time in women, it increased for men. The most prevalent type of eating disorder was OSFED, which also increased with age, suggesting that the DSM-5 criteria better capture classical eating disorders in younger men, and fail to reflect how disordered eating presents in aged individuals [14].

Mitchell *et al.*[15^▪^] surveyed US military veterans using three different eating disorder measurement tools, the Eating Disorder Diagnostic Scale-5 (EDDS-5), the EDE-Q, and the SCOFF (Sick, Control, One, Fat and Food) questionnaire, and found that a considerable percentage of male participants had disordered eating behavior on all three measures (EDDS-5 – 8.5%, EDE-Q – 25.2%, SCOFF – 11.1%). Rates of probable eating disorders were significantly higher in 40–49 years old participants compared with other age groups. Although rates on all three measures were roughly half of those in women, the numbers are still considerable and in line with reports from our and other groups.

Finally, while Hilbert *et al.*[53] showed a peak of EDE-Q total scores not in young men but in the age group 55–64 years, a Canadian study showed that men aged 45–49 years showed the highest EDE-Q total scores compared with the other age groups investigated, although this difference did not reach statistical significance [13].

### Associated factors

In addition to the influences already discussed, two new associations with eating disorders in aging men are worth mentioning.

We found in our community sample of Austrian men, that individuals scoring high on the Aging Males’ Symptoms (AMS) scale showed higher prevalence rates of eating disturbances than low-scoring men [54]. The AMS scale is a tool to assess symptoms of aging [55], and high scores on the AMS scale have been shown to be indicative of a possible testosterone deficiency [56]. An age-related decline in testosterone could, therefore, be responsible for men developing eating disorders or associated symptoms at older age (see [57^▪^] for a review of this topic).

Hooper *et al.*[58^▪^] investigated the association between negative body talk and eating disorders. While they found that engagement with critical and negative talk about weight-related body issues (i.e. fat talk) did show strong correlations with eating disorder pathology and body dissatisfaction, especially in men aged 40 and older, this was not the case for negative body talk focused on changes due to aging (i.e. old talk). Engagement with fat talk was also associated with stronger depressive symptoms. As aging alone was associated with lower body dissatisfaction, promoting body acceptance in older men could, therefore, contribute to an overall increase in well being and help prevent the development of eating disorder pathology.

## CONCLUSION

Eating disorders do occur in middle and older age of both sexes; shame and stigmatization have decreased and medical awareness of explicit assessment of eating behavior in all age groups has developed. Existing data show that it is a complex process when new diagnoses enter new age cohorts, such as the recognition of eating disorders among older people. There is still a lack of treatment studies, because they are always at the very end of scientific assessments, after the knowledge of the new disorder has arrived in the public perception. What puberty is for eating disorders in adolescence and young age is menopausal transition for midlife women. Also in men, associations with hormonal disturbances are possible. Treatment approaches should consider treatment strategies tailored to older women and men, addressing the context of midlife and aging.

## Acknowledgements

*The authors would like to thank Judith Offringa for her editorial assistance.*

### Financial support and sponsorship

*None.*

### Conflicts of interest

*There are no conflicts of interest.*

## REFERENCES AND RECOMMENDED READING

Papers of particular interest, published within the annual period of review, have been highlighted as:

▪ of special interest
▪▪ of outstanding interest
